# Toxin Fused with SUMO Tag: A New Expression Vector Strategy to Obtain Recombinant Venom Toxins with Easy Tag Removal inside the Bacteria

**DOI:** 10.3390/toxins9030082

**Published:** 2017-02-27

**Authors:** Lhiri H. A. L. Shimokawa-Falcão, Maria C. Caporrino, Katia C. Barbaro, Maisa S. Della-Casa, Geraldo S. Magalhães

**Affiliations:** Laboratory of Immunopathology, Butantan Institute, Av. Vital Brazil 1500, 05503-900 São Paulo, SP, Brazil; lhiri.hanna@gmail.com (L.H.A.L.S.-F.); maria.caporrino@butantan.gov.br (M.C.C.); katia.barbaro@butantan.gov.br (K.C.B.); maisa.casa@butantan.gov.br (M.S.D.-C.)

**Keywords:** construction vector, venom toxin, phospholipase D, *Loxosceles gaucho*, SUMO tag, disintegrins Ulp1 protease, snake toxin, spider toxin, protein expression

## Abstract

Many animal toxins may target the same molecules that need to be controlled in certain pathologies; therefore, some toxins have led to the formulation of drugs that are presently used, and many other drugs are still under development. Nevertheless, collecting sufficient toxins from the original source might be a limiting factor in studying their biological activities. Thus, molecular biology techniques have been applied in order to obtain large amounts of recombinant toxins into *Escherichia coli*. However, most animal toxins are difficult to express in this system, which results in insoluble, misfolded, or unstable proteins. To solve these issues, toxins have been fused with tags that may improve protein expression, solubility, and stability. Among these tags, the SUMO (small ubiquitin-related modifier) has been shown to be very efficient and can be removed by the Ulp1 protease. However, removing SUMO is a labor- and time-consuming process. To enhance this system, here we show the construction of a bicistronic vector that allows the expression of any protein fused to both the SUMO and Ulp1 protease. In this way, after expression, Ulp1 is able to cleave SUMO and leave the protein interest-free and ready for purification. This strategy was validated through the expression of a new phospholipase D from the spider *Loxosceles gaucho* and a disintegrin from the *Bothrops insularis* snake. Both recombinant toxins showed good yield and preserved biological activities, indicating that the bicistronic vector may be a viable method to produce proteins that are difficult to express.

## 1. Introduction

Although venoms are essentially used for defense and/or prey immobilization, they represent a natural reservoir of several biologically active molecules that might become a valuable tool for the development of new biotechnological and pharmaceutical products [1,2,3,4]. Furthermore, understanding how toxins work will also assist in the development of better treatment for human envenomation [5]. However, obtaining enough toxins from the original source is one of the major challenges in this field. To overcome this problem, many toxins genes have been expressed into heterologous organisms to obtain sufficient material to perform functional, structural, and biochemical characterization. Among the systems available to express recombinant proteins, *Escherichia coli* is by far the first choice due to its ease of manipulation, inexpensive culture, and rapid growth [6,7].

As spiders produce a limited amount of venom, this system has been of extreme value to study interesting toxins with antiarrhythmic [8], antimicrobial [9], analgesic [10], insecticidal [11] and haemolytic activities [12]. Snake toxins have also been expressed into *E. coli* and this approach has allowed the characterization of toxins with anti-thrombotic [13], anticancer [14], anti-inflammatory [15], antimicrobial activities [16] as well as fused toxins for the development of serotherapy against envenomation [17]. However, when a recombinant protein is synthesized in *E. coli*, the microenvironment is different from that of the original source, which may lead to protein aggregation, known as inclusion bodies [18]. To solve this problem, some peptide/protein tags are incorporated into the protein of interest (POI) to increase expression yields and influence solubility and native folding. In this regard, the expression of many toxins was only possible after its fusion to these tags. The most common and best-performing tags for this purpose are glutathione S-transferase (GST) [19], Nus A [20], thioredoxin (Trx) [21], maltose binding protein (MBP) [22] and small ubiquitin-related modifier (SUMO) [23]. Among these tags, it has been shown that SUMO presents superior advantages to enhance the expression and solubility of the POI [24].

SUMO belongs to a group of ubiquitin-like proteins around 11 kDa that are highly conserved in eukaryotes cells and the mostly used by being fused to the N-terminal of the POI to increase its expression and solubility [25]. SUMO can be removed from the POI by the Ulp1 protease that recognizes only SUMO tertiary structure and leaves the POI with its native N-terminal, making this system more efficient than other tags [26]. While the use of SUMO fusion technology has several advantages, the procedure of removing it from the POI is still a labor-intensive process, mainly due to the necessity to express and purify the Ulp1 protease. In addition, the reaction to cleave SUMO from POI takes about four to 16 h.

To exploit the SUMO expression system and resolve some drawbacks associated with it, this work shows the modification of a commercial bicistronic vector to allow both the expression of the POI fused to SUMO and the Ulp1 protease. In this way, Ulp1 will remove SUMO from the POI right inside the bacteria, leaving it ready for purification in a single expression step, making it a much faster and simpler process. This system was validated through the expression of LgRec2, a new phospholipase D cloned from the venom gland of *Loxosceles gaucho*. This molecule belongs to a family of genus-specific phospholipase D (PLD), which is responsible for the main toxic effects of envenomations by *Loxosceles* sp. [27]. Despite the cloning of several PLD isoforms from *L. intermedia* [28,29,30,31,32,33] and *L. laeta* [34,35,36,37] into *E. coli*, LgRec2 is only the second PLD cloned and expressed from *L. gaucho*.

The vector was also tested through the expression of Insularin, a disintegrin isolated from the viper *Bothrops insularis* that previously could not be expressed without a tag [38]. This class of toxins from viper venoms shows great potential to develop new anti-thrombotic [39] and antitumoral agents [40], but these molecules have been difficult to express in *E. coli* because of their high cysteine content [41,42]. Using a specially designed expression vector, we demonstrate below the successful expression of both a novel spider PLD and a snake disintegrin into *E. coli*.

## 2. Results

### 2.1. Construction of the pSUMO_Ulp1_ Vector

**Figure 1:**
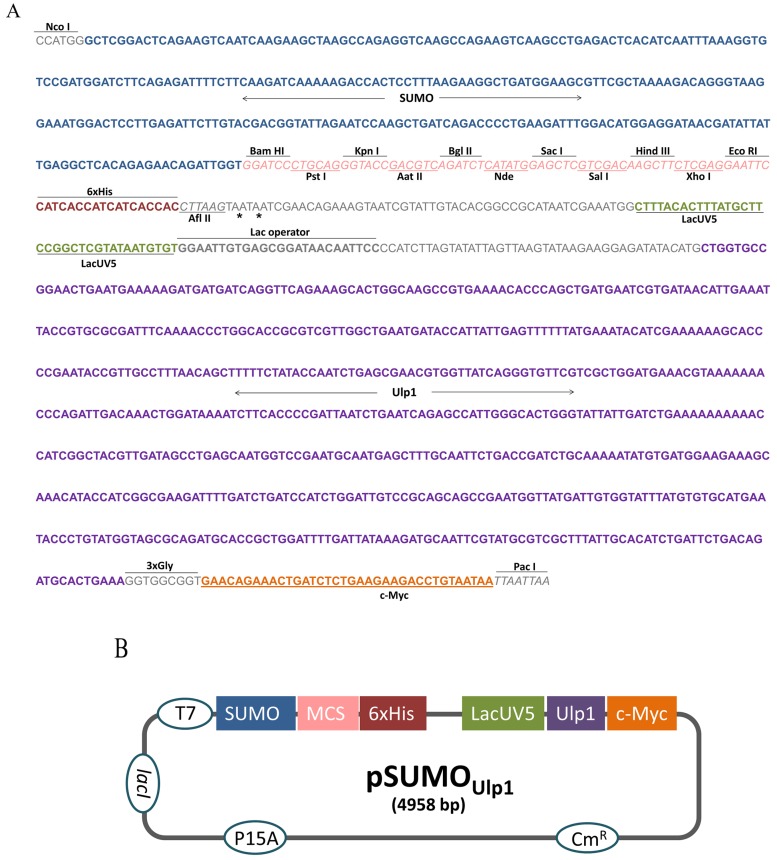
pSUMO_Ulp1_ vector construction. (**A**) Sequence of the cassette designed to perform vector construction, asterisks indicate stop codon; (**B**) Schematic diagram of the vector developed in this study. The elements inserted into the backbone vector pACYCDuet-1 are shown in the boxes as follows: SUMO (small ubiquitin-related modifier); MCS (multiple policloning site); 6xHis (six histidine tag); LacUV5 (promoter); Ulp1 (SUMO protease); and c-Myc (polypeptide protein tag derived from the c-Myc gene). The elements already present in pACYCDuet-1 vector are shown in the circles as follows: T7 (promoter); Cm^R^ (chloramphenicol resistance gene); lacI (regulatory gene for lac operon); and P15A (origin of replication).

To construct the pSUMO_Ulp1_ vector, the region between the restriction enzymes *Nco* I and *Pac* I of the bicistronic pACYCDuet-1 vector was removed and replaced by a synthetic DNA insert (Figure 1A). This insert contained, in the following order, the SUMO sequence to facilitate protein expression and solubility, a polycloning site for cloning and a six histidines tag (6xHis tag) for purification by Ni-affinity chromatography. In order to remove the SUMO from the protein of interest (POI), the sequence of Ulp1 protease under control of *lacUV5* promoter was also inserted into the cassette. Furthermore, a c-Myc tag was placed at the end of the Ulp1 sequence for detection purposes. Three glycines were added after Ulp1 to ensure c-Myc tag flexibility and enhance its detection.

In this construction, the POI fused to the SUMO and Ulp1 protease is expressed at the same time, which allows the removal of the SUMO tag inside the bacteria. Figure 1B shows the schematic representation of the vector that was constructed.

### 2.2. Evaluation of Ulp1 and SUMO Expression

**Figure 2:**
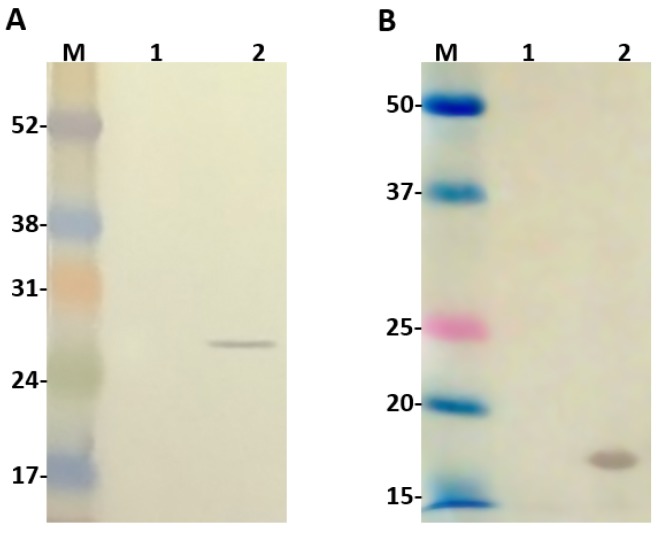
Western blot analysis 1 and 2: extract from BL21 Star™ (DE3) cells before and after isopropyl-b-d-thiogalactoside (IPTG) induction, respectively. (**A**) Ulp1 protease detected with anti-c-Myc monoclonal antibody; (**B**) SUMO detected with Anti-SUMO polyclonal antibody. Numbers on the left correspond to position of molecular mass markers (M) in kilodaltons.

The second promoter T7, originaly present in pACYCDuet-1 vector, was replaced by a weaker one (*lacUV*5) to guide the expression of Ulp1. For this reason, before conducting any cloning in this vector, we decided to evaluate the expression of Ulp1. For this, the vector constructed was transformed into the BL21 *Star*™ (DE3) chemically competent cells and their extract, with or without IPTG induction, was analyzed by western blot using anti-c-Myc monoclonal antibodies, since this tag is available at the C-terminus of Ulp1. As seen (Figure 2A), Ulp1 was recognized on the induced IPTG extract with an expected molecular size of 25.5 kDa. The same analysis was also performed to identify SUMO expression and one band of expected molecular size around 16 kDa was identified by the anti-SUMO polyclonal antibodies on the bacteria extract induced with IPTG (Figure 2B).

The second promoter T7, originaly present in pACYCDuet-1 vector, was replaced by a weaker one (*lacUV*5) to guide the expression of Ulp1. For this reason, before conducting any cloning in this vector, we decided to evaluate the expression of Ulp1. For this, the vector constructed was transformed into the BL21 *Star*™ (DE3) chemically competent cells and their extract, with or without IPTG induction, was analyzed by western blot using anti-c-Myc monoclonal antibodies, since this tag is available at the C-terminus of Ulp1. As seen (Figure 2A), Ulp1 was recognized on the induced IPTG extract with an expected molecular size of 25.5 kDa. The same analysis was also performed to identify SUMO expression and one band of expected molecular size around 16 kDa was identified by the anti-SUMO polyclonal antibodies on the bacteria extract induced with IPTG (Figure 2B).

### 2.3. Cloning of LgRec2 from L. gaucho Venom Gland and Alignment Analysis

**Figure 3:**
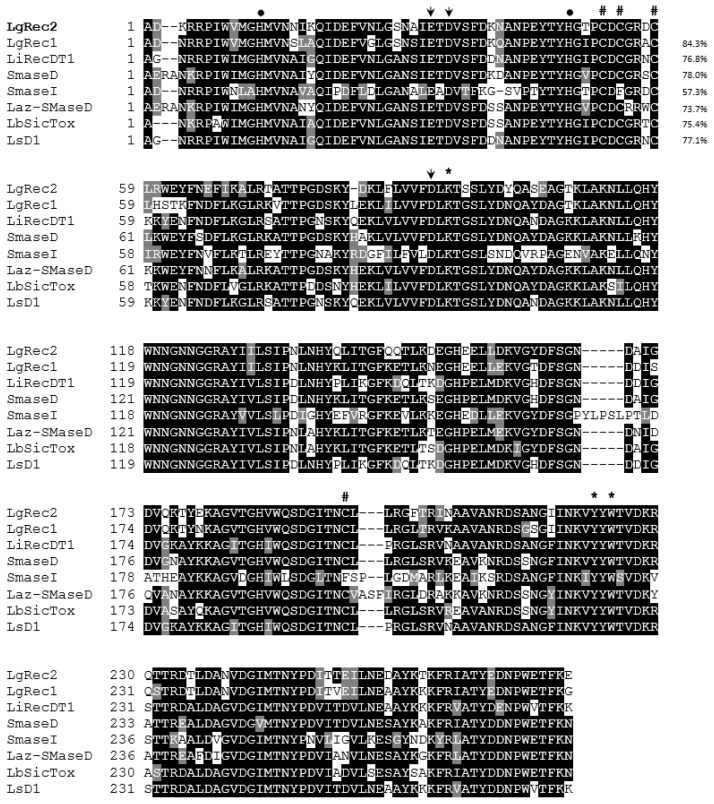
Multiple alignment analysis of deduced amino acid sequences of mature LgRec2 (KY192527), LgRec1 (K9USW8) from *L. gaucho*, LiRecDT1 (P0CE80) from *L. intermedia*, SMase D (P0CE79) from *L. reclusa*, SMase I (Q8I914) from *L. laeta*, Laz-SMaseD (Q4ZFU2) from *L. arizonica*, LbSicTox (Q5YD77) from *L. boneti* and LsD1 (Q56JA9) from *L. similis*. The percentages on the right indicate the identity shared between LgRec2 and other phospholipases D from the genus *Loxosceles*. Dark and gray shaded regions show identical and conserved amino acids, respectively. The conserved cysteines are indicated by hash symbols. The Mg^2+^ binding site and the two catalytic histidines that form the catalytic site are indicated by arrows and dots, respectively. The residues possibly involved in substrate recognition are indicated by asterisks. Sequences alignment and shading were performed with T-Coffee and BoxShade, respectively.

Previously, our group cloned the first phospholipase D (LgRec1) from *L. gaucho* using the 3′RACE system (Thermofisher) and a degenerated 5′ primer that was designed based on the alignment of phospholipases D from *L. laeta*, *L. intermedia* and *L. reclusa* [43]. By using the same technique, we were able to isolate a new phospholipase D sequence that was named LgRec2. Its sequence was deposited in the GenBank under accession number KY192527. The predicted mature amino acid sequence of LgRec2 was shown to be comprised of 279 amino acids with a predicted molecular mass of 31,851 Da and a pI of 5.20. Multiple alignments of LgRec2 amino acids sequenced with LgRec1 and other phospholipases D cloned from different *Loxosceles genus* are shown in Figure 3. Considering the high identity of LgRec2 among the sequences and previous works on the crystal structure of recombinant phospholipases D Smase I from *L. laeta* [44] and LiRecDT1 from *L. intermedia* [45], it is possible to infer the location of the two catalytic histidines (dots), the Mg^2+^ binding site (arrows) and the residues possibly involved in substrate recognition (asterisks). Still based on these works, phospholipase D members were divided into class I or II if they possessed one or two disulfide bridges, respectively. Therefore, LgRec2 belongs to class II and shows the highest identity (84.3%) with LgRec1 and the lowest (57.3%) with Smase I from *L. laeta*, which is a class I due to the mutation C201F.

### 2.4. Expression of LgRec2 Using pSUMO_Ulp1_ Vector

**Figure 4:**
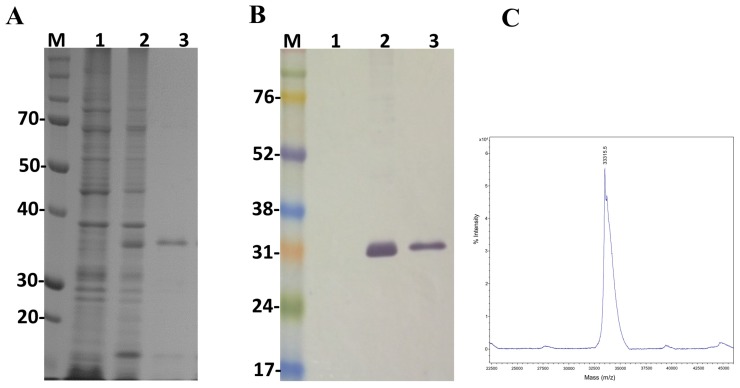
Characterization of the recombinant toxin LgRec2 overexpressed in *E. coli* BL21 Star™ (DE3) using pSUMO_Ulp1_ vector. Numbers on the left correspond to position of molecular mass markers (M) in kDa. 1 and 2: extract from BL21 Star™ (DE3) before and after IPTG induction, respectively; 3: purified/dialyzed LgRec2. (**A**) SDS-PAGE analysis of recombinant LgRec2 expression under reduction conditions stained with Coomassie blue; (**B**) Western blot analysis with monoclonal anti-polyHistidine antibody; (**C**) MALDI-TOF MS analysis of purified LgRec2. The spectra were acquired in positive ion linear mode using default calibration as described in Materials and Methods.

In order to study the main biological activities of LgRec2, we tried to express this toxin using the same vector and procedures conducted on LgRec1 [43]. However, despite several attempts using different temperatures and IPTG (Isopropyl β-d-1-thiogalactopyranoside) concentrations, it was not possible to obtain LgRec2 in soluble form (data not shown). Therefore, the sequence of LgRec2 was cloned into the pSUMO_Ulp1_ vector as the presence of SUMO may have facilitated its expression and solubility. Next, this construction was transformed into *BL21 Star*™ (DE3) chemically competent cells and the expression was performed at 30 °C for four hours. Sonicated bacteria supernatant was then purified by Ni^2+^-affinity chromatography and analyzed by SDS-PAGE. The electrophoretic profile showed a band around 35 kDa (Figure 4A). However, when analyzed by mass spectrometry it revealed a molecular mass of 33,315 Da (Figure 4C), a little higher than the predicted mass due to the presence of the 6xHis tag. The expression was also analyzed by western blot using anti-His tag antibodies. As seen in Figure 4B, the recombinant LgRec2 was recognized in both induced and purified fractions. The overall yield of LgRec2 was about 24 mg of purified protein recovered per liter of cell culture.

### 2.5. Analysis of Biological Activities of LgRec2

**Figure 5:**
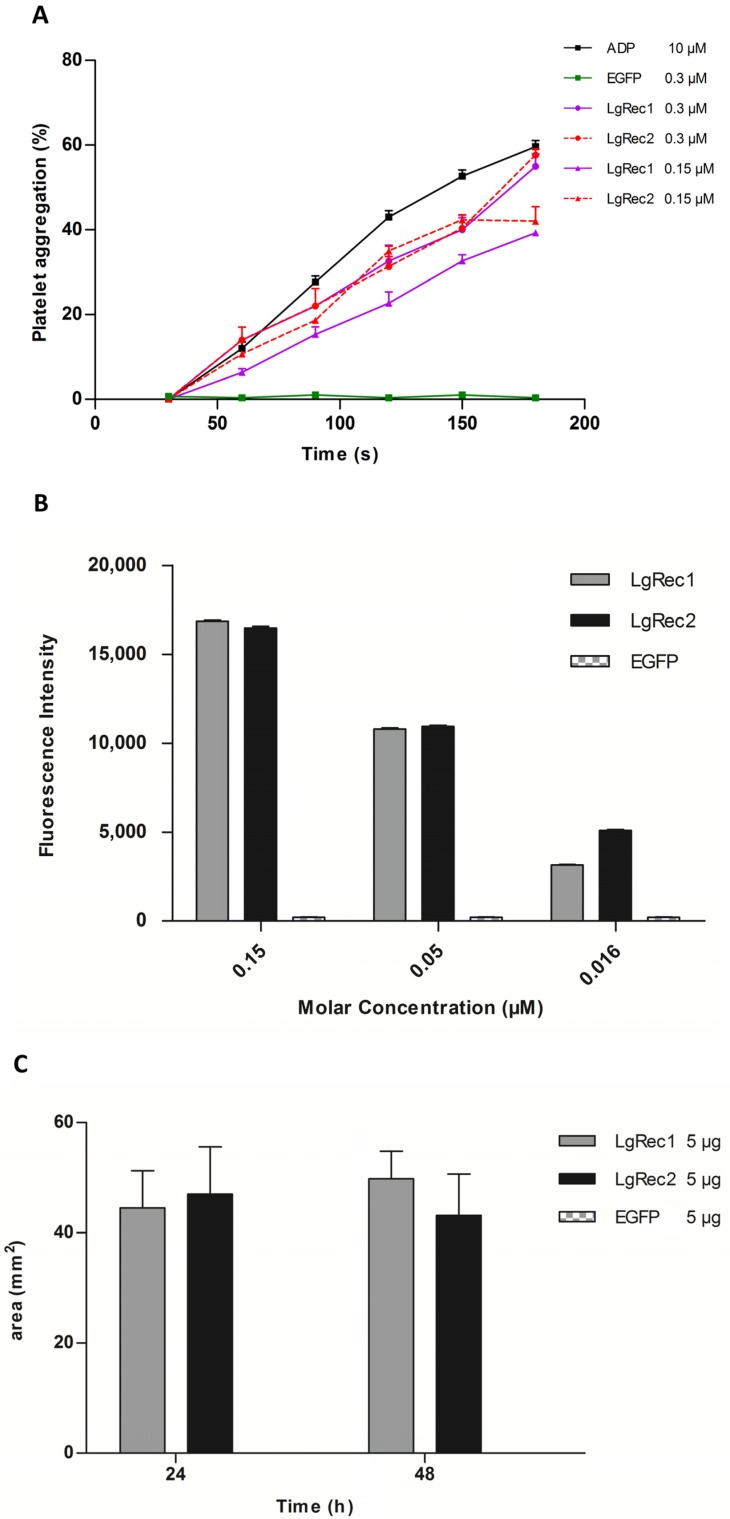
Biological characterization of the recombinant toxin LgRec2. (**A**) Effect of LgRec2 on platelet aggregation. Platelet-rich plasma was incubated with 0.3 or 0.15 μM of LgRec2, LgRec1 (positive control) or EGFP (negative control). Aggregation was monitored by measuring the light transmittance for three minutes by an aggregometer (*n* = 3). The extent of the induction of platelet aggregation was assessed by comparison with the maximal aggregation induced by the control dose of agonist (10 μM ADP); (**B**) Sphingomyelinase activities of the recombinant dermonecrotic toxins (0.15; 0.05 and 0.016 μM) LgRec2, LgRec1 and the negative control EGFP were evaluated with the Amplex Red Sphingomyelinase Assay Kit; (**C**) Dermonecrosis was induced by intradermal injection of 0.2 mL of PBS containing 5 μg of purified LgRec2, LgRec1 (positive control) or EGFP (negative control) into the rabbit dorsum (*n* = 3). The local reaction areas (mm^2^) were measured 24 and 48 h after the injection (*n* = 3). Values given are the average ± SEM.

After the successful expression of LgRec2 free from SUMO, its main biological activities were tested. First, its property to promote platelet aggregation was evaluated as it is an important activity of phospholipases D isolated from *Loxosceles* [46]. In this test, the platelets viability was first evaluated with ADP (adenosine diphosphate). Then LgRec1 as well as a non-related protein (EGFP) were used as a positive and negative control, respectively. The analysis showed that while LgRec1 exhibited a small decrease in platelet aggregation when concentration was decreased by half, LgRec2 still maintained its activity at both concentrations (Figure 5A). Next, LgRec2 was assayed for its ability to cleave sphingomyelin, as this is also an intrinsic characteristic of this class of toxins [47]. As shown in Figure 5B, both LgRec2 and LgRec1 exhibited concentration-dependent activity. Considering that *Loxosceles* phospholipases D can cause dermonecrotic lesions in mammalian tissues, LgRec2 was injected into shaved rabbit skin and the areas of dermonecrosis were measured. As seen in Figure 5C, both LgRec2 and LgRec1 exhibited lesion areas at 24 h and 48 h. In all the experiments, EGFP did not show any activity. There was no statistically significant difference in these both activities for LgRec2 and LgRec1.

### 2.6. Expression of Insularin Using the pSUMO_Ulp1_ Vector

**Figure 6:**
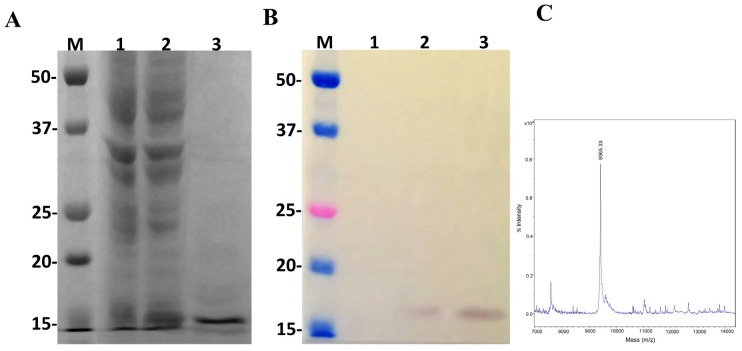
Characterization of the recombinant disintegrin Insularin (INS) overexpressed in *E. coli* BL21 Star™ (DE3) using the pSUMO_Ulp1_ vector. Numbers on the left correspond to position of molecular mass markers (M) in kDa. 1 and 2: extract from BL21 Star™ (DE3) before and after IPTG induction, respectively; 3: purified/dialyzed INS. (**A**) 15% SDS-PAGE analysis of INS expression under reduction conditions and stained with Coomassie blue. (**B**) Western blot analysis with monoclonal anti-polyHistidine antibody. (**C**) MALDI-TOF MS analysis of purified Insularin. The spectra were acquired in positive ion linear mode using the default calibration as described in Section 5.

Once the pSUMO_Ulp1_ vector showed to be effective to express LgRec2 and remove SUMO from it, we decided to test this vector further by cloning the sequence of the disintegrin isolated from the venom gland of *Bothrops insularis*. Insularin (INS) was chosen as this toxin contains a high number of cysteines and its expression was only possible in fusion with glutathione S-transferase [38]. pSUMO_Ulp1_-INS was transformed into *BL21 Star*™ (DE3) cells and the expression was performed at 30 °C for four hours. As shown in Figure 6A, a band around 16 kDa was visualized in SDS-PAGE (Polyacrylamide Gel Electrophoresis with Sodium Dodecyl Sulfate) but when analyzed by mass spectrometry, it showed a molecular mass of 9365.33 Da (Figure 6C), which corresponded to the expected size of this molecule with 6xHis tag. In addition, western blot using anti-His tag monoclonal antibody was only able to recognize free Insularin (Figure 6B). The average yield of Insularin was 20 mg per liter of cell culture.

### 2.7. Analysis of Platelet Inhibition Activity of Insularin

**Figure 7:**
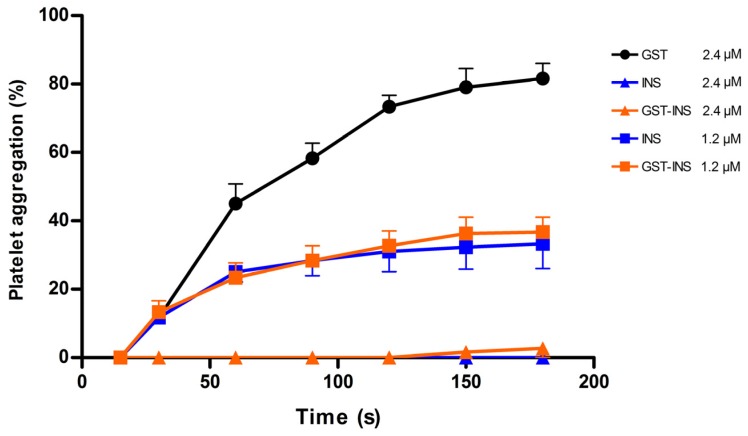
Effect of recombinant Insularin (INS) and Insularin fused to GST (GST-INS) on inhibition of platelet aggregation. Platelet-rich plasma was incubated with different concentrations of INS, GST-INS or GST that was used as negative control. Platelet aggregation was induced by adding 10 μM ADP. Aggregation was monitored by measuring the light transmittance for three minutes by an aggregometer (*n* = 3). Values given are the average ± SEM.

Insularin is a disintegrin with a high affinity for integrins such as α_IIb_β_3_ present on platelets. Once Insularin binds to this receptor, it is able to inhibit platelet aggregation upon ADP challenge. Therefore, an aggregation assay using human platelets was carried out with both Insularin and Insularin fused to GST (GST-INS) for comparison purposes. The results (Figure 7) showed that both proteins presented inhibition of aggregation at doses of 1.2 μM (~50%) and 2.4 μM (~100%) with no statistically significant difference after ADP addition, indicating that Insularin was as active as GST-INS. The GST, used as a negative control, showed no effect on platelet inhibition.

## 3. Discussion

The use of molecular biology techniques has contributed significantly to the knowledge of animal toxins [48,49,50,51], and have provided means in which to obtain large amounts of these molecules in order to perform biological characterization. *Escherichia coli* has been the work-horse of gene expression for many years, but unfortunately, many recombinant toxins have been expressed as inclusion bodies [14,52,53,54,55], requiring exhaustive in vitro refolding steps to access their biological activities.

In attempting to solve this problem, fusion proteins have been employed to increase the solubility and stability of many proteins of interest (POI). In our experience, SUMO were very effective at expressing a single chain variable fragment (scFv) antibody against a venom metalloproteinase BaP1 from the viper *Bothrops asper* [56] and a disintegrin from the viper *Bothrops neuwiedi* [57], both of which were previously obtained only as inclusion bodies. However, the process of removing SUMO is laborious and time-consuming. In order to enhance this system, this paper presents the construction of the pSUMO_Ulp1_ vector. In this construction, the 6x His tag sequence was placed after the polycloning site so that the POI will have the 6x His tag at its C-terminus, which will avoid the purification of any POI that suffered from premature termination. To remove SUMO from the POI, the sequence of the Ulp1 protease (that recognizes only the tertiary structure of SUMO [58]) was also inserted into this vector.

Considering that the Ulp1 protease was very efficient, requiring a ratio of 1:1000 enzyme to the substrate [26], a weaker promoter (*lacUV5*) was used to drive its expression so the bacteria were not overloaded with unnecessary exogenous protein. At the C-terminus of Ulp1, a c-Myc tag sequence was inserted to detect the expression of this protease, as it does not contain a tag for purification. As seen in the western blot analysis, the *lacUV5* promoter was efficient at expressing the Ulp1 protease in sufficient amounts necessary to remove SUMO from the POI inside the bacteria.

In previous studies, we cloned LgRec1 [43], the first phospholipase D (PLD) isolated from the venom of *L. gaucho*; in this work, we applied the same technique to present the cloning of another PLD named LgRec2. It has been shown that isoforms of PLDs from the genus *Loxosceles* are the main toxins present in the venom and are of great importance, since they are responsible for most of the toxic effects observed in loxoscelism, such as platelet aggregation, dermonecrosis, hemolysis, nephrotoxicity, and inflammatory response [46,59]. In addition, PLDs are also able to promote the hydrolysis of glycerophospholipids or sphingophospholipids, generating phosphatidic acid, lysophosphatidic acid, ceramide 1-phosphate and choline [60], of which the biological functions remain unknown. Therefore, the biological characterization of isoforms from this class of toxins is very important in understanding how their mechanism of action may help develop better treatments for envenomations.

The amino acids alignment of LgRec2 showed high identity with other PLDs from *Loxosceles*, including the cysteines and catalytic site residues. Despite sharing high identity with LgRec1, LgRec2 could not be expressed in soluble form, probably because LgRec2 is slightly hydrophobic, which among other factors, might promote the insolubility of some proteins [61]. Therefore, to solubilize LgRec2, its sequence was cloned into the pSUMO_Ulp1_ vector. The expression was conducted at 30 °C to decrease the protein synthesis rate, which usually produces a higher yield of soluble and correctly folded protein [62]. This temperature does not pose a problem for Ulp1 cleavage as it is active over a wide range of buffer and temperature conditions [26]. The results showed that the pSUMO_Ulp1_ vector was very efficient at expressing the LgRec2 and Ulp1 protease because SUMO was completely removed from LgRec2, and only a single band, corresponding to LgRec2, was detected in the western blot.

To test if LgRec2 was properly folded and active, its ability to induce platelet aggregation, to hydrolyze sphingomyelin and to promote dermonecrosis in rabbit skin was analyzed. In these experiments, LgRec2 was assayed along with LgRec1 to evaluate possible activity variations between both PLDs, as studies comparing recombinant PLD isoforms from *Loxosceles* have shown differences in the intensity of their effects [30,31,37,63]. In all experiments, LgRec2 showed similar activities to LgRec1, except for platelet aggregation for which LgRec2 was slightly more active. Possible explanations for these similarities include their identical catalytic histidines (His^12^ and His^48^), their substrate recognition residues (K^94^, Y^223^ and W^225^), and the Mg^2+^ binding site (E^32^, D^34^ and D^92^) which is essential for catalysis [45]. Although it was suggested that differences in hydrophobicity at the boundaries of the catalytic site of *Loxosceles* PLDs were responsible for differentiated toxic effects, this was not the case for LgRec2 in relation to LgRec1 [64]. Despite their similar activities, the differences in their amino acid composition (~16%) might translate into different effects during envenomation or specificity for many species preyed by *L. gaucho*. After all, feeding and defense have been an important evolutionary force leading to toxin diversification.

As disulfide bond formation in the cytoplasm may lead to protein inactivation, misfolding and aggregation [64], we decided to further evaluate the potential of the vector pSUMO_Ulp1_ by expressing Insularin, a cysteine-rich (~16%) disintegrin isolated from the venom gland of *Bothrops insularis*. Disintegrins are cysteine-rich peptides from viper venoms that contain a tripeptide RGD motif that binds with high-affinity to integrins receptors. Therefore, these molecules can interfere in important processes involved in carcinogenesis, tumor growth and invasion, which makes them interesting molecules for cancer treatment [40]. In platelets, they can inhibit aggregation by competitively blocking fibrinogen binding to α_IIb_β_3_ receptors, representing an important tool as anti-thrombotic agents [13].

Previously, the expression of Insularin was only possible after its fusion with glutathione S-transferase (GST) [38]. However, the GST could not be removed, probably because the small thrombin cleavage site was sterically hindered. In our experiments using the pSUMO_Ulp1_ vector, Insularin was expressed in soluble form and free from the SUMO tag, as shown in the western blot analysis. Although displaying a migration around 16 kDa on SDS-PAGE, mass spectrometry analysis revealed that its molecular mass was 9.6 kDa. This abnormal gel migration has also been reported with other disintegrins [65,66] and some other proteins [67]. The reason for this gel shifting is still unclear, but research indicates that some proteins seem to bind differently on SDS, thus appearing to be larger or smaller than expected [68]. When analyzed, recombinant Insularin showed complete inhibitory activity upon ADP-induced platelet aggregation at 1.2 μM. In addition, its activity was very similar to GST-INS, indicating that the GST tag was not diminishing the binding of Insularin to platelets integrins. Therefore, in this case, the removal of GST may not be mandatory, but the expression and purification of GST to be used as a negative control represents a time-consuming process.

## 4. Conclusions

The expression vector used in this work allowed the production of two recombinant toxins with complex characteristics of structure/function with preserved biological activities. Therefore, the data presented here suggest that the pSUMO_Ulp1_ vector may be an efficient tool for obtaining recombinant toxins and other proteins that are difficult to express. In addition, its unique tag removal approach saves time and costs involved in the process of getting the protein of interest-free from SUMO.

## 5. Materials and Methods

### 5.1. Construction of the pSUMO_Ulp1_ Vector

A DNA fragment containing in this order, the nucleotide sequences of the SUMO protein (Genbank: NM_001180818); a multiple polycloning site; six histidine; a Lac UV5 promoter; Ulp1_403–621_ (amino acids 403–621) protease (Genbank: NM 001183834); three glycines; and a sequence of a c-Myc tag was synthesized by Invitrogen™ Gene Synthesis (GeneArt™), Thermo Fisher Scientific, Waltham, MA, USA. This fragment, along with pACYCDuet™-1 (Novagen^®^ Merck Corporation, Darmstadt, Germany) vector were digested with *Nco* I and *Pac* I restriction enzymes. Next, the digestion products were purified with a PureLink^®^ quick gel extraction kit (Thermo Fisher Scientific, Waltham, MA, USA) following the provided protocol. After this process, both vector and insert were joined by T4 DNA ligase (New England Biolabs Inc, Ipswich, MA, USA) and used to transform One Sho^t®^ TOP10 chemically competent *E. coli* (Thermo Fisher Scientific, Waltham, MA, USA) . The transformed bacteria were then plated on LB-Agar containing chloramphenicol (25 µg/mL) and resistant colonies were PCR (Polymerase Chain Reaction) screened for the insert. The PCR positive colonies were submitted to plasmid extraction followed by automatic sequencing using an ABI PRISM^®^ 373 DNA Sequencer (Thermo Fisher Scientific, Waltham, MA, USA). The construction was named pSUMO_Ulp1_.

### 5.2. Cloning of LgRec2 and INS into pSUMO_Ulp1_ Vector

The coding sequence of phospholipase D LgRec2 from *Loxosceles gaucho* spider was obtained using degenerate primers designed from the alignment of phospholipase’s cDNAs previously described for other species of *Loxosceles*. The entire process to isolate the LgRec2 sequence follows the protocol previously described [43]. Both the PCR-amplified LgRec2 and pSUMO_Ulp1_ vector were digested with *Bam*HI and *Hind*III, purified and submitted to ligation, generating the pSUMO_Ulp1_LgRec2 vector.

The fragment of the cDNA encoding for the Insularin disintegrin (INS) from *Bothrops insularis* (Genbank: AY736107) was amplified by high fidelity PCR using a previous construction called pGST-INS [38] as a template. The primers were designed to contain a *Bam*HI and *Xho*I restriction sites at 5´and 3´ends, respectively. The PCR products as well as pSUMO_Ulp1_ were digested with *Xho*I and *Bam*HI enzymes, purified and submitted to ligation, resulting in the pSUMO_Ulp1_INS vector.

### 5.3. Protein Expression and Purification

Chemically competent *E. coli* BL21 Star™ (DE3) (Invitrogen^®^) were transformed with pSUMO_Ulp1_LgRec2 or pSUMO_Ulp1_INS vectors. For every experiment, an overnight grown cell colony from LB-agar plates was transferred into liquid LB medium and grown overnight at 30 °C in the presence of 25 µg/mL chloramphenicol. This culture was diluted 1:50 into 200 mL of fresh LB broth/chloramphenicol for small scale purification. The cell suspension was allowed to reach an optical density between 0.5 and 0.6 at 30 °C (OD_600_ nm) before the addition of isopropyl-b-d-thiogalactoside (IPTG) at 1 mM final concentration. Cells were then grown for four hours at 30 °C. After the induction time, the cells were collected by centrifugation at 5000 g for 10 min at 4 °C and either immediately used or stored frozen at −20 °C.

For purification, whole-cell pellets were resuspended in binding buffer (50 mM sodium phosphate, 300 mM NaCl, pH 7.0). The cells were lysed by an ultrasonication device on ice for 60 s for intervals of two minutes for cooling, with total sonication time of 10 min. Cell debris were removed from the protein solution by centrifugation at 10,000 g for 10 min. The entire amount of the supernatant containing the soluble protein was purified by immobilized metal affinity chromatography (IMAC) using Ni Sepharose^®^ 6 Fast Flow GE^®^ (GE Healthcare, Little Chalfont, UK) following the manufacturer´s recommendations. The protein was dialyzed against standard phosphate-buffered saline (PBS) buffer. Quantification of recombinant toxins was performed using the BCA Pierce™ Protein Assay Kit (Thermo Fisher Scientific, Waltham, MA, USA) following the manufacturer´s protocol.

### 5.4. Determination of Molecular Mass by Mass Spectrometry

This procedure was carried out by the Center for Research Support (CEFAP) of the Institute of Biomedical Sciences (ICB) of the University of São Paulo (USP). Analyses were performed on a MALDI-TOF^®^ (Bruker Corporation, Billerica, MA, USA) following pre-established protocols for protein analysis. The procedure consisted of the application of 0.5 μL of a saturated solution of sinapinic acid in ethanol on the samples of INS and LgRec2. After drying, 1 μL of INS and LgRec2 were mixed with 1 μL of a TA30 saturated solution (0.1% trifluoroacetic acid/acetonitrile, 70:30) and finally 1 μL was applied to the Ground Steel plate for analysis. Data acquisition was performed in linear mode with positive polarity, with the following parameters: Ion Source 1—19.50 kV, Ion Source 2—17.60 kV, Lens—9.0 kV, Pulsed Ion Extraction 170 ns, Mass Range 5–70 kDa, Laser Frequency 500 Hz, Gain Detector 10.0 x. The results were analyzed using the online software mMass version 5.5.0, 2013, (Martin Strohalm^©^ Open Source Mass Spectrometry Tool, www.mmass.org).

### 5.5. SDS-Polyacrylamide Gel Electrophoresis and Western Blot Analysis

Sample of the recombinants purified proteins (5 μg) or bacteria culture samples before and after IPTG induction were analyzed by SDS-PAGE 15% under reducing (2.5% dithiothreitol) conditions. After electrophoresis, proteins were stained with Coomassie Blue R-250 or transferred onto nitrocellulose membranes.

Nitrocellulose membrane transfers were performed using the Trans-Blot^®^ SD Semi-Dry Transfer Cell (Bio-Rad Laboratories, Hercules, CA, USA) following the manufacturer´s recommendations. After transfer, the nitrocellulose membranes were stained with Ponceau S^®^ (Merck Millipore Corporation, Darmstadt, Germany) at 1:20 dilution to verify transfer efficiency. To remove the dye, the membranes were washed with TBS-Tween (20 mM Tris, 150 mM NaCl, 0.05% Tween 20, pH 7.5) until complete removal. The membranes were incubated for two hours with rabbit polyclonal anti-SUMO antibody at 1:4000 dilution, mouse monoclonal anti-polyHistidine antibody (Sigma Life Science) or mouse monoclonal anti-c-Myc (Sigma Life Science, Merck Corporation, Darmstadt, Germany) at 1:1000 dilution. Membranes were washed with TBS-Tween and immunoreactive proteins were detected using 1:500 diluted peroxidase-labeled anti-rabbit IgG (Sigma Life Science), or anti-mouse IgG (Sigma Life Science) and the blot was revealed with 4-chloro-1-naphthol.

To infer the molecular mass, the following standards were used: Amersham RainbowMarker—Full range^™^ GE^®^ (GE Healthcare, Little Chalfont, UK), PageRuler™ Prestained Protein Ladder (Thermo Fisher Scientific, Waltham, MA, USA) or the Dual Xtra Prestained Protein™ Standards (Bio-Rad Laboratories, Hercules, CA, USA).

### 5.6. Sphingomyelinase Activity

Sphingomyelinase activity was measured using the Amplex^®^ Red Assay Kit (Thermo Fisher Scientific, Waltham, MA, USA) following the manufacturer’s recommendations. This assay evaluates, by an indirect method, the capacity of the phospholipase D to hydrolyze the phosphodiester bond of sphingomyelin. The recombinant proteins LgRec2, LgRec1 (positive control) [43] and the enhanced green fluorescent protein (EGFP) as a negative control were added to the Amplex^®^ Red reagent mixture at doses of 0.15, 0.05 and 0.016 μM each in three trials. The plate containing the samples (100 μL) was incubated at 37 °C for 30 min, before fluorescence was measured in a spectrofluorometer Spectramax M2 (Molecular Devices LLC., Sunnyvale, CA, USA) using excitation at 540 nm and emission detection at 590 nm.

### 5.7. Platelet Aggregation Assay

Human blood was collected in a 3.8% sodium citrate solution (1:9), after informed consent, from healthy volunteers who declared not to have taken medications known to interfere with platelet function for the previous 10 days (São Paulo Health Institute-Ethical Committee for Human Protocol: 019/2008). Platelet aggregation using platelet rich plasma (PRP) was carried out as previously described [69]. In the inhibition assays involving the disintegrins, aliquots of 0.4 mL of PRP were incubated at 37 °C with Insularin (INS); glutathione S-transferase (GST), used as a negative control; or Insularin fused with GST (GST-INS) [38] as a positive control, at concentrations of 2.4 and 1.2 μM for three minutes prior to the stimulation of platelet aggregation by 10 μM ADP (Sigma Life Science). In the aggregation assay involving LgRec2, LgRec1 or EGFP at concentrations of 0.3 μM and 0.15 μM, proteins were incubated with 0.4 mL of PRP at 37 °C with no need of ADP stimulation. Platelet poor plasma (PPP) was used as a blank. The extent of the inhibition or induction of platelet aggregation was assessed by comparing it with the maximal aggregation induced by the control dose of agonist (10 μM ADP). The experiments were performed in triplicate (*n* = 3) in a Chrono-log aggregometer, model 490 (Chrono-Log Corporation, Havertown, PA, USA).

### 5.8. Dermonecrotic Activity

The procedures involving animals were conducted according to national laws and policies controlled by the Butantan Institute Animal Investigation Ethical Committee (protocol No 886/12). Local dermonecrotic reaction activity was assayed by intradermal injection of 0.2 mL of PBS containing 5 μg of purified LgRec2, LgRec1 (positive control) or EGFP (negative control), into a shaved area of rabbit dorsal flank. The dermonecrotic areas were examined 24 and 48 h after injection and reported as the average ± SEM of the areas in mm^2^ (*n* = 3).

### 5.9. Statistical Analyses

Statistical analyses were performed using analysis of variance (ANOVA) with the post-hoc Tukey test in the GraphPad Prism 5 software version 5.01, 2007. (GraphPad Software, Inc. La Jolla, CA, USA). Significance was considered when *p* ≤ 0.05.

## References

[1] Chaisakul J., Hodgson W.C., Kuruppu S., Prasongsook N. (2016). Effects of animal venoms and toxins on hallmarks of cancer. J. Cancer.

[2] De Marco V., Stier G., Blandin S., de Marco A. (2004). The solubility and stability of recombinant proteins are increased by their fusion to nusa. Biochem. Biophys. Res. Commun..

[3] Senff-Ribeiro A., da Silva P.H., Chaim O.M., Gremski L.H., Paludo K.S., da Silveira R.B., Gremski W., Mangili O.C., Veiga S.S. (2008). Biotechnological applications of brown spider (*loxosceles genus*) venom toxins. Biotechnol. Adv..

[4] Menez A. (1998). Functional architectures of animal toxins: A clue to drug design?. Toxicon.

[5] Chaves-Moreira D., de Moraes F.R., Caruso Í.P., Chaim O.M., Senff-Ribeiro A., Ullah A., Da Silva L.S., Chahine J., Arni R.K., Veiga S.S. (2016). Potential implications for designing drugs against the brown spider venom phospholipase-D. J. Cell. Biochem..

[6] Gopal G.J., Kumar A. (2013). Strategies for the production of recombinant protein in *Escherichia coli*. Protein J..

[7] Rosano G.L., Ceccarelli E.A. (2014). Recombinant protein expression in *Escherichia coli*: Advances and challenges. Front. Microbiol..

[8] Almeida A.P., Andrade A.B., Ferreira A.J., Pires A.C.G., Damasceno D.D., Alves M.N.M., Gomes E.R.M., Kushmerick C., Lima R.F., Prado M.A.M. (2011). Antiarrhythmogenic effects of a neurotoxin from the spider *phoneutria nigriventer*. Toxicon.

[9] Shlyapnikov Y.M., Andreev Y.A., Kozlov S.A., Vassilevski A.A., Grishin E.V. (2008). Bacterial production of latarcin 2a, a potent antimicrobial peptide from spider venom. Protein Expr. Purif..

[10] Sermadiras I., Revell J., Linley J.E., Sandercock A., Ravn P. (2013). Recombinant expression and in vitro characterisation of active Huwentoxin-IV. PLoS ONE.

[11] Bende N.S., Dziemborowicz S., Herzig V., Ramanujam V., Brown G.W., Bosmans F., Nicholson G.M., King G.F., Mobli M. (2015). The insecticidal spider toxin SFI1 is a knottin peptide that blocks the pore of insect voltage-gated sodium channels via a large beta-hairpin loop. FEBS J..

[12] Chaves-Moreira D., Souza F.N., Fogaca R.T.H., Mangili O.C., Gremski W., Senff-Ribeiro A., Chaim O.M., Veiga S.S. (2011). The relationship between calcium and the metabolism of plasma membrane phospholipids in hemolysis induced by brown spider venom phospholipase-D toxin. J. Cell. Biochem..

[13] Huang T.F., Hsu C.C., Kuo Y.J. (2016). Anti-thrombotic agents derived from snake venom proteins. Thromb. J..

[14] Lyukmanova E.N., Shulepko M.A., Shenkarev Z.O., Kasheverov I.E., Chugunov A.O., Kulbatskii D.S., Myshkin M.Y., Utkin Y.N., Efremov R.G., Tsetlin V.I. (2016). Central loop of non-conventional toxin WTX from *Naja kaouthia* is important for interaction with nicotinic acetylcholine receptors. Toxicon.

[15] Sun D.S., Ho P.H., Chang H.H. (2016). Soluble P-selectin rescues viper venom-induced mortality through anti-inflammatory properties and PSGL-1 pathway-mediated correction of hemostasis. Sci. Rep..

[16] Yamane E.S., Bizerra F.C., Oliveira E.B., Moreira J.T., Rajabi M., Nunes G.L.C., de Souza A.O., da Silva I., Yamane T., Karpel R.L. (2013). Unraveling the antifungal activity of a south american rattlesnake toxin crotamine. Biochimie.

[17] Ducancel F., Boulain J.C., Tremeau O., Menez A. (1989). Direct expression in Escherichia coli of a functionally active protein A-snake toxin fusion protein. Protein Engineering.

[18] Carrio M.M., Villaverde A. (2001). Protein aggregation as bacterial inclusion bodies is reversible. FEBS Lett..

[19] Zhang H., Huang P.F., Meng E., Li W.Y., Zhou L., Zhu L.Y., Wu L., Li M.J., Liang S.P., Zhang D.Y. (2015). An efficient strategy for heterologous expression and purification of active peptide Hainantoxin-IV. PLoS ONE.

[20] Jiang X.P., Xu J.Q., Yang Q. (2010). Soluble expression, purification, and characterization of gloydius shedaoensis venom gloshedobin in *Escherichia coli* by using fusion partners. Appl. Microbiol. Biotechnol..

[21] Yuan S.L., Duan H.Q., Liu C.J., Liu X.L., Liu T.T., Tao H.X., Zhang Z.S. (2004). The role of thioredoxin and disulfide isomerase in the expression of the snake venom thrombin-like enzyme calobin in *Escherichia coli* BL21 (DE3). Protein Expr. Purif..

[22] Vu T.T.T., Jeong B., Yu J., Koo B.K., Jo S.H., Robinson R.C., Choe H. (2014). Soluble prokaryotic expression and purification of crotamine using an n-terminal maltose-binding protein tag. Toxicon.

[23] Hernandez-Cuebas L.M., White M.M. (2012). Expression of a biologically-active conotoxin PrIIIE in *Escherichia coli*. Protein Expr. Purif..

[24] Marblestone J.G., Edavettal S.C., Lim Y., Lim P., Zuo X., Butt T.R. (2006). Comparison of sumo fusion technology with traditional gene fusion systems: Enhanced expression and solubility with sumo. Protein Sci..

[25] Bird L.E. (2011). High throughput construction and small scale expression screening of multi-tag vectors in *Escherichia coli*. Methods.

[26] Malakhov M., Mattern M., Malakhova O., Drinker M., Weeks S., Butt T. (2004). Sumo fusions and sumo-specific protease for efficient expression and purification of proteins. J. Struct. Funct. Genom..

[27] Gremski L.H., Trevisan-Silva D., Ferrer V.P., Matsubara F.H., Meissner G.O., Wille A.C.M., Vuitika L., Dias-Lopes C., Ullah A., de Moraes F.R. (2014). Recent advances in the understanding of brown spider venoms: From the biology of spiders to the molecular mechanisms of toxins. Toxicon.

[28] Kalapothakis E., Araujo S.C., de Castro C.S., Mendes T.M., Gomez M.V., Mangili O.C., Gubert I.C., Chavez-Olortegui C. (2002). Molecular cloning, expression and immunological properties of LiD1, a protein from the dermonecrotic family of, *Loxosceles intermedia* spider venom. Toxicon.

[29] Chaim O.M., Sade Y.B., da Silveira R.B., Toma L., Kalapothakis E., Chavez-Olortegui C., Mangili O.C., Gremski W., von Dietrich C.P., Nader H.B. (2006). Brown spider dermonecrotic toxin directly induces nephrotoxicity. Toxicol. Appl. Pharmacol..

[30] da Silveira R.B., Pigozzo R.B., Chaim O.M., Appel M.H., Dreyfuss J.L., Toma L., Mangili O.C., Gremski W., Dietrich C.P., Nader H.B. (2006). Molecular cloning and functional characterization of two isoforms of dermonecrotic toxin from *Loxosceles intermedia* (brown spider) venom gland. Biochimie.

[31] da Silveira R.B., Pigozzo R.B., Chaim O.M., Appel M.H., Silva D.T., Dreyfuss J.L., Toma L., Dietrich C.P., Nader H.B., Veiga S.S. (2007). Two novel dermonecrotic toxins LiRecDT4 and LiRecDT 5 from brown spider (*Loxosceles intermedia*) venom: From cloning to functional characterization. Biochimie.

[32] Appel M.H., da Silveira R.B., Chaim O.M., Paludo K.S., Silva D.T., Chaves D.M., da Silva P.H., Mangili O.C., Senff-Ribeiro A., Gremski W. (2008). Identification, cloning and functional characterization of a novel dermonecrotic toxin (phospholipase D) from brown spider (*Loxosceles intermedia*) venom. Biochim. Biophys. Acta Gen. Subj..

[33] Vuitika L., Gremski L.H., Belisario-Ferrari M.R., Chaves-Moreira D., Ferrer V.P., Senff-Ribeiro A., Chaim O.M., Veiga S.S. (2013). Brown spider phospholipase-D containing a conservative mutation (D233E) in the catalytic site: Identification and functional characterization. J. Cell. Biochem..

[34] Catalan A., Cortes W., Sagua H., Gonzalez J., Araya J.E. (2011). Two new phospholipase D isoforms of *Loxosceles laeta*: Cloning, heterologous expression, functional characterization, and potential biotechnological application. J. Biochem. Mol. Toxicol..

[35] Pedrosa M.D., de Azevedo I.D., Goncalves-de-Andrade R.M., van den Berg C.W., Ramos C.R.R., Ho P.L., Tambourgi D.V. (2002). Molecular cloning and expression of a functional dermonecrotic and haemolytic factor from *Loxosceles laeta* venom. Biochem. Biophys. Res. Commun..

[36] Tambourgi D.V., Pedrosa M.D.F., van den Berg C.W., Goncalves-de-Andrade R.M., Ferracini M., Paixao-Cavalcante D., Morgan B.P., Rushmere N.K. (2004). Molecular cloning, expression, function and immunoreactivities of members of a gene family of sphingomyelinases from *Loxosceles* venom glands. Mol. Immunol..

[37] Ferrara G.I.D., Fernandes-Pedrosa M.D., Junqueira-de-Azevedo I.D.M., Goncalves-de-Andrade R.M., Portaro F.C.V., Manzoni-de-Almeida D., Murakami M.T., Arni R.K., van den Berg C.W., Ho P.L. (2009). Smase II, a new sphingomyelinase D from *Loxosceles laeta* venom gland: Molecular cloning, expression, function and structural analysis. Toxicon.

[38] Della-Casa M.S., Junqueira-de-Azevedo I., Butera D., Clissa P.B., Lopes D.S., Serrano S.M.T., Pimenta D.C., Magalhães G.S., Ho P.L., Moura-da-Silva A.M. (2011). “Insularin, a disintegrin from *Bothrops insularis* venom: Inhibition of platelet aggregation and endothelial cell adhesion by the native and recombinant gst-insularin proteins”. Toxicon.

[39] Huang S.W., Kuo H.L., Hsu M.T., Tseng Y.J., Lin S.W., Kuo S.C., Peng H.C., Lien J.C., Huang T.F. (2016). A novel thromboxane receptor antagonist, nstpbp5185, inhibits platelet aggregation and thrombus formation in animal models. Thromb. Haemost..

[40] Macedo J.K.A., Fox J.W., Castro M.D. (2015). Disintegrins from snake venoms and their applications in cancer research and therapy. Curr. Protein Pept. Sci..

[41] Carey C.M., Bueno R., Gutierrez D.A., Petro C., Lucena S.E., Sanchez E.E., Soto J.G. (2012). Recombinant rubistatin (r-Rub), an MVD disintegrin, inhibits cell migration and proliferation, and is a strong apoptotic inducer of the human melanoma cell line SK-Mel-28. Toxicon.

[42] Suntravat M., Barret H.S., Jurica C.A., Lucena S.E., Perez J.C., Sánchez E.E. (2015). Recombinant disintegrin (r-Cam-dis) from *Crotalus adamanteus* inhibits adhesion of human pancreatic cancer cell lines to laminin-1 and vitronectin. J. Venom Res..

[43] Magalhães G.S., Caporrino M.C., Della-Casa M.S., Kimura L.F., Prezotto-Neto J.P., Fukuda D.A., Portes J.A., Neves-Ferreira A.G.C., Santoro M.L., Barbaro K.C. (2013). Cloning, expression and characterization of a phospholipase D from *Loxosceles gaucho* venom gland. Biochimie.

[44] Murakami M.T., Fernandes-Pedrosa M.F., Tambourgi D.V., Arni R.K. (2005). Structural basis for metal ion coordination and the catalytic mechanism of sphingomyelinases D. J. Biol. Chem..

[45] de Giuseppe P.O., Ullah A., Silva D.T., Gremski L.H., Wille A.C.M., Moreira D.C., Ribeiro A.S., Chaim O.M., Murakami M.T., Veiga S.S. (2011). Structure of a novel class II phospholipase D: Catalytic cleft is modified by a disulphide bridge. Biochem. Biophys. Res. Commun..

[46] Tavares F.L., Peichoto M.E., Rangel D.D., Barbaro K.C., Cirillo M.C., Santoro M.L., Sano-Martins I.S. (2011). *Loxosceles gaucho* spider venom and its sphingomyelinase fraction trigger the main functions of human and rabbit platelets. Hum. Exp. Toxicol..

[47] Lajoie D.M., Roberts S.A., Zobel-Thropp P.A., Delahaye J.L., Bandarian V., Binford G.J., Cordes M.H.J. (2015). Variable substrate preference among phospholipase D toxins from *Sicariid* spiders. J. Biol. Chem..

[48] Brahma R.K., McCleary R.J.R., Kini R.M., Doley R. (2015). Venom gland transcriptomics for identifying, cataloging, and characterizing venom proteins in snakes. Toxicon.

[49] Hodgson D., Gasparini S., Drevet P., Ducancel F., Bouet F., Boulain J.C., Harris J.B., Menez A. (1993). Production of recombinant notechis 11’2l, an enzymatically active mutant of a phospholipase-a2 from notechis-scutatus scutatus venom, as directly generated by cleavage of a fusion protein produced in *Escherichia coli*. Eur. J. Biochem..

[50] Bouchier C., Ducancel F., Guignery-Frelat G., Bon C., Boulain J.C., Ménez A. (1988). Cloning and sequencing of cDNAs encoding the two subunits of Crotoxin. Nucleic Acids Res..

[51] Ducancel F., Guigneryfrelat G., Boulain J.C., Menez A. (1990). Nucleotide-sequence and structure-analysis of cDNAs encoding short-chain neurotoxins from venom glands of a sea-snake (*Aipysurus laevis*). Toxicon.

[52] Balduino K.N., Spencer P.J., Malavasi N.V., Chura-Chambi R.M., Lemke L.S., Morganti L. (2011). Refolding by high pressure of a toxin containing seven disulfide bonds: Bothropstoxin-1 from *Bothrops jararacussu*. Mol. Biotechnol..

[53] Dai H., Yin S.J., Li T., Cao Z.J., Ji Y.H., Wu Y.L., Li W.X. (2012). Recombinant expression, purification, and characterization of scorpion toxin BmαTX14. Protein Expr. Purif..

[54] Estrada G., Silva A.O., Villegas E., Ortiz E., Beirao P.S.L., Corzo G. (2016). Heterologous expression of five disulfide-bonded insecticidal spider peptides. Toxicon.

[55] Ferrer V.P., de Mari T.L., Gremski L.H., Silva D.T., da Silveira R.B., Gremski W., Chaim O.M., Senff-Ribeiro A., Nader H.B., Veiga S.S. (2013). A novel hyaluronidase from brown spider (*Loxosceles intermedia*) venom (Dietrich’s hyaluronidase): From cloning to functional characterization. PLoS Negl. Trop. Dis..

[56] Castro J.M.A., Oliveira T.S., Silveira C.R.F., Caporrino M.C., Rodriguez D., Moura-Da-Silva A.M., Ramos O.H.P., Rucavado A., Gutierrez J.M., Magalhães G.S. (2014). A neutralizing recombinant single chain antibody, scFv, against BAP1, a P-I hemorrhagic metalloproteinase from *Bothrops asper* snake venom. Toxicon.

[57] Lima-Dos-Santos I., Della-Casa M.S., Portes J.A., Calabria P.A.L., Magalhães G.S., Moura-da-Silva A.M. (2015). Characterization of neuwiedin, a new disintegrin from *Bothrops neuwiedi* venom gland with distinct cysteine pattern. Toxicon.

[58] Mossessova E., Lima C.D. (2000). Ulp1-SUMO crystal structure and genetic analysis reveal conserved interactions and a regulatory element essential for cell growth in yeast. Mol. Cell.

[59] Barbaro K.C., Sousa M.V., Morhy L., Eickstedt V.R.D., Mota I. (1996). Compared chemical properties of dermonecrotic and lethal toxins from spiders of the genus *Loxosceles* (*araneae*). J. Protein Chem..

[60] Lee S., Lynch K.R. (2005). Brown recluse spider (*Loxosceles reclusa*) venom phospholipase D (PLD) generates lysophosphatidic acid (LPA). Biochem. J..

[61] Idicula-Thomas S., Balaji P.V. (2005). Understanding the relationship between the primary structure of proteins and its propensity to be soluble on overexpression in *Escherichia coli*. Protein Sci..

[62] Terpe K. (2006). Overview of bacterial expression systems for heterologous protein production: From molecular and biochemical fundamentals to commercial systems. Appl. Microbiol. Biotechnol..

[63] Ribeiro R.O.S., Chaim O.M., da Silveira R.B., Gremski L.H., Sade Y.B., Paludo K.S., Senff-Ribeiro A., de Moura J., Chavez-Olortegui C., Gremski W. (2007). Biological and structural comparison of recombinant phospholipase D toxins from *Loxosceles intermedia* (brown spider) venom. Toxicon.

[64] Derman A.I., Prinz W.A., Belin D., Beckwith J. (1993). Mutations that allow disulfide bond formation in the cytoplasm of *Escherichia coli*. Science.

[65] Fernandez J.H., Silva C.A., Assakura M.T., Camargo A.C.M., Serrano S.M.T. (2005). Molecular cloning, functional expression, and molecular modeling of Bothrostatin, a new highly active disintegrin from *Bothrops jararaca* venom. Biochem. Biophys. Res. Commun..

[66] Yeh C.H., Peng H.C., Yih J.B., Huang T.F. (1998). A new short chain RGD-containing disintegrin, accutin, inhibits the common pathway of human platelet aggregation. Biochim. Biophys. Acta Gen. Subj..

[67] Halder T., Agarwal T., Ray S. (2016). Isolation, cloning, and characterization of a novel *Sorghum dehydrin* (SbDhn2) protein. Protoplasma.

[68] Rath A., Glibowicka M., Nadeau V.G., Chen G., Deber C.M. (2009). Detergent binding explains anomalous SDS-PAGE migration of membrane proteins. Proc. Natl. Acad. Sci. USA.

[69] Santoro M.L., Sano-Martins I.S. (2004). Platelet dysfunction during *Bothrops jararaca* snake envenomation in rabbits. Thromb. Haemost..

